# Structural analysis of the LDL receptor–interacting FERM domain in the E3 ubiquitin ligase IDOL reveals an obscured substrate-binding site

**DOI:** 10.1074/jbc.RA120.014349

**Published:** 2020-07-29

**Authors:** Luca Martinelli, Athanassios Adamopoulos, Patrik Johansson, Paul T. Wan, Jenny Gunnarsson, Hongwei Guo, Helen Boyd, Noam Zelcer, Titia K. Sixma

**Affiliations:** 1 Division of Biochemistry, Netherlands Cancer Institute, Amsterdam, The Netherlands; 2 Department of Medical Biochemistry, Amsterdam UMC, Amsterdam Cardiovascular Sciences and Gastroenterology and Metabolism, University of Amsterdam, Amsterdam, the Netherlands; 3 IMED Biotech Unit, Discovery Sciences, AstraZeneca, Mölndal, Sweden; 4 Oncode Institute, Utrecht, The Netherlands

**Keywords:** crystal structure, E3 ubiquitin ligase, ubiquitylation (ubiquitination), cholesterol metabolism, IDOL, MYLIP, LDLR, FERM, lipoprotein metabolism, low-density lipoprotein (LDL), protein structure, small-angle X-ray scattering (SAXS), enzyme purification, FERM domain, LDL receptor

## Abstract

Hepatic abundance of the low-density lipoprotein receptor (LDLR) is a critical determinant of circulating plasma LDL cholesterol levels and hence development of coronary artery disease. The sterol-responsive E3 ubiquitin ligase inducible degrader of the LDLR (IDOL) specifically promotes ubiquitination and subsequent lysosomal degradation of the LDLR and thus controls cellular LDL uptake. IDOL contains an extended N-terminal FERM (4.1 protein, ezrin, radixin, and moesin) domain, responsible for substrate recognition and plasma membrane association, and a second C-terminal RING domain, responsible for the E3 ligase activity and homodimerization. As IDOL is a putative lipid-lowering drug target, we investigated the molecular details of its substrate recognition. We produced and isolated full-length IDOL protein, which displayed high autoubiquitination activity. However, *in vitro* ubiquitination of its substrate, the intracellular tail of the LDLR, was low. To investigate the structural basis for this, we determined crystal structures of the extended FERM domain of IDOL and multiple conformations of its F3ab subdomain. These reveal the archetypal F1-F2-F3 trilobed FERM domain structure but show that the F3c subdomain orientation obscures the target-binding site. To substantiate this finding, we analyzed the full-length FERM domain and a series of truncated FERM constructs by small-angle X-ray scattering (SAXS). The scattering data support a compact and globular core FERM domain with a more flexible and extended C-terminal region. This flexibility may explain the low activity *in vitro* and suggests that IDOL may require activation for recognition of the LDLR.

Hepatic low-density lipoprotein receptor (LDLR) activity is a central determinant of circulating levels of LDL cholesterol (1), elevation of which represents a major risk factor for development of coronary artery disease. Therapeutic approaches that increase hepatic abundance of the LDLR (*e.g.* by use of statins) form the cornerstone of cholesterol-lowering strategies in hypercholesterolemic individuals (2). In view of its central role in lipoprotein metabolism, the levels, and hence activity, of the LDLR are subject to tight transcriptional regulation by the sterol-regulatory element–binding proteins (3–5). Next to transcriptional regulation, the importance of post-transcriptional control of LDLR abundance has gained recognition in recent years (6). Two central post-transcriptional pathways have been implicated in controlling LDLR abundance through regulated degradation of the receptor. The first depends on the secreted protein proprotein convertase subtilisin/kexin type 9 (PCSK9), which binds to the LDLR ectodomain and directs the normally recycling receptor toward lysosomal degradation (7–10). Targeting of this pathway through the use of anti-PCSK9 antibodies that sequester PCSK9 and prevent its interaction with the LDLR has been proven highly effective in lowering LDL cholesterol in humans (10, 11). The second pathway involves the sterol-regulated inducible degrader of the LDLR (IDOL, also known as MYLIP), which is an E3 ubiquitin ligase that promotes the ubiquitylation and subsequent lysosomal degradation of the LDLR (6, 12, 13).

Two distinct protein domains are present in IDOL: an N-terminal FERM (band 4.1/ezrin/radixin/moesin) and a C-terminal RING domain (12, 14). In contrast to secreted PCSK9, which interacts with the LDLR extracellularly, IDOL is a 45-kDa cytoplasmic protein that interacts with the intracellular tail of the LDLR via its FERM domain (12). Recognition of the intracellular tail of the LDLR is mediated by the FERM domain at the plasma membrane, and structural homology modeling facilitated the identification of a putative helix that coordinates the interaction between IDOL and a conserved motif within the intracellular tail of the LDLR (14). A similar function for the FERM domain in mediating protein-protein and membrane-protein interactions has been reported already for several other FERM domain–containing proteins (15, 16). The RING domain is needed for recruitment of the E2 ubiquitin-conjugating enzyme to IDOL, and both UBC13 and the UBE2D family of E2s have been demonstrated to work in concert with IDOL to promote sterol-dependent degradation of the LDLR (14, 17). In addition to its role in lipid metabolism, IDOL has also been reported to trigger the ubiquitination and degradation of the very-low-density lipoprotein receptor and ApoER2 (two closely related LDLR family members), suggesting a possible role of IDOL in neuronal development and function (13, 18–20). Importantly, despite both culminating in lysosomal degradation of the LDLR, the endocytic route followed by ubiquitylated LDLR is distinct from that used by PCSK9 (21); IDOL-mediated degradation of the LDLR is clathrin- and ARH (aryl hydrocarbon receptor)-independent and requires sorting through the ESCRT (endosomal sorting complexes required for transport) system (22, 23). As such, IDOL represents an alternative and complementary post-transcriptional pathway to that governed by PCSK9 to modulate LDLR abundance.

Therapeutic targeting of IDOL, like that of PCSK9 is supported by genome-wide association studies that identified an association between variation in the *IDOL/MYLIP* locus and circulating LDL levels in humans (24). Further supporting this notion, we recently identified carriers of the first loss-of-function *IDOL* variant (25), the presence of which was associated with reduced circulating levels of LDL cholesterol. Yet targeting of IDOL may extend beyond cholesterol-lowering regimens; silencing of *Idol* expression in mice using antisense RNA protects mice from Alzheimer-like pathology (19, 26). Additionally, we recently reported that absence of *Idol* improves their glucose handling and protects *Idol^ko^* mice from dyslipidemia, obesity, and hepatosteatosis during normal aging and when challenged with a Western-type diet (27). Hence, IDOL inhibition may be broadly beneficial in metabolic-syndrome and aging-associated morbidities. However, the lack of a high-resolution structure of IDOL and a detailed description of how it interacts with the intracellular tail of the LDLR hampers development of therapeutic modalities to inhibit its activity. Here we present *in vitro* analysis of IDOL enzymatic activity showing limited LDLR peptide ubiquitination and a structural analysis of IDOL's FERM domain exploring this.

## Results

### Purification and characterization of recombinant full-length IDOL

**Figure 1.**
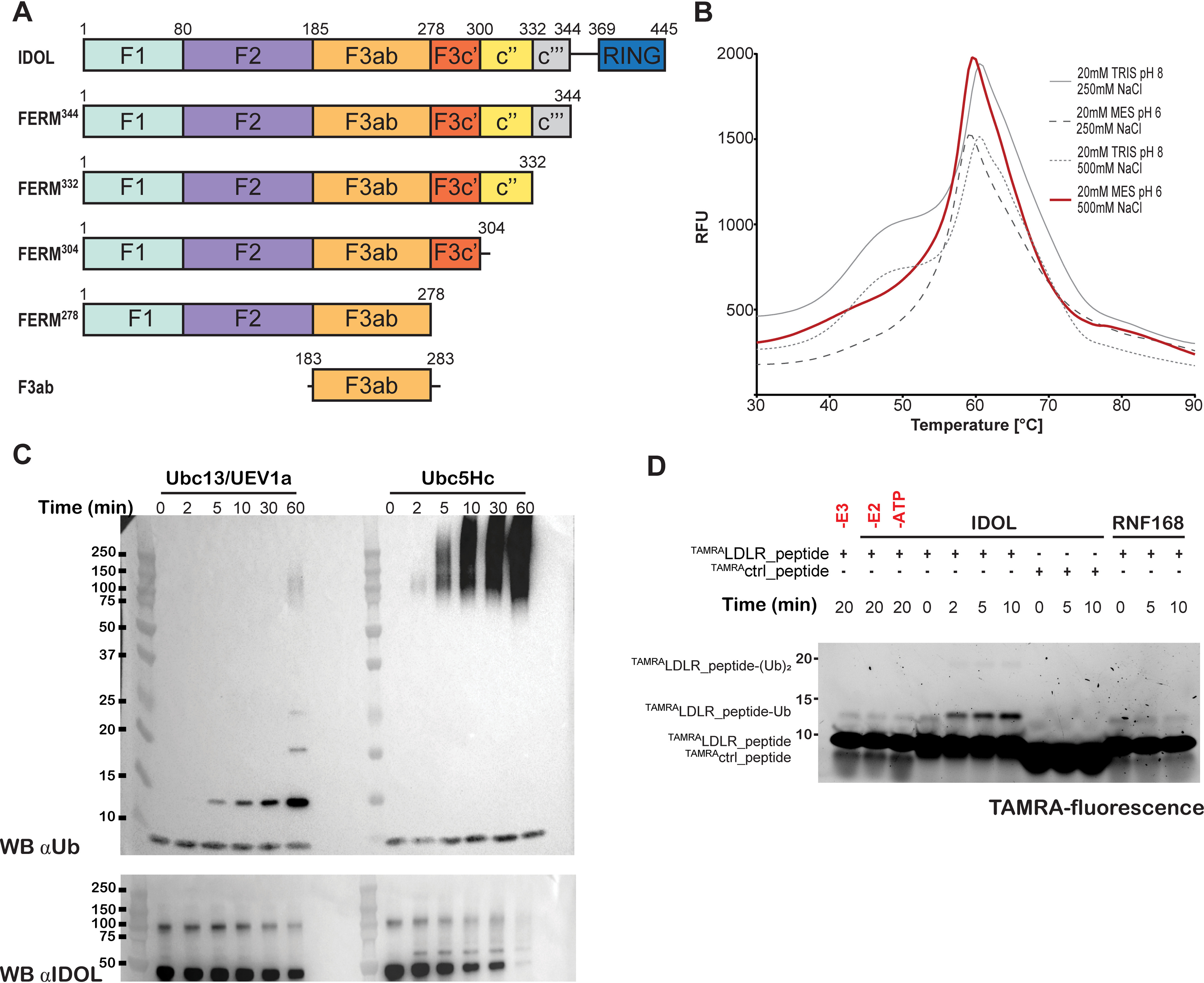
**Characterization full-length IDOL.** *A*, *schematic view* of the IDOL constructs used in this study. *Numbers* indicate amino acid positions. Subdomain boundaries are indicated. *B*, melting curves reflecting temperature-dependent IDOL conformational stability for full-length IDOL under different conditions. Results are shown in relative fluorescence units (*RFU*). All buffers contained 1 mm TCEP. *C*, *in vitro* autoubiquitination (*top*) and ubiquitin polychain formation (*bottom*) IDOL activity assay. IDOL autoubiquitination was detected by immunoblotting for IDOL, whereas ubiquitin polychain formation was detected by immunoblotting (*WB*) for ubiquitin. *D*, *in vitro* IDOL-dependent LDLR_peptide ubiquitination. TAMRA fluorescence signals were acquired by a ChemiDoc XRS+ imaging system (Bio-Rad).

Full-length IDOL consists of 445 amino acids, organized in two functional domains: an N-terminal FERM domain and a C-terminal RING domain (Fig. 1*A*) (12, 14, 28). To study IDOL ligase activity and its structure, recombinant full-length human IDOL protein was purified from insect cells.

We noticed that size-exclusion chromatography (SEC) profiles of human IDOL were characterized by multiple peaks, suggesting different oligomerization/aggregation states (Fig. S1). We then analyzed by differential scanning fluorimetry (DSF), also known as ThermoFluor, whether the IDOL purification profile was influenced by a combination of different buffer and salt conditions. In most conditions, this resulted in a profile characterized by a double transition, suggesting heterogeneity (Fig. 1*B* and Fig. S1), whereas only at low pH (pH 4–6) and intermediate NaCl concentration (500–750 mm), IDOL melting curves are characterized by a single transition (*red line* in Fig. 1*B* and Fig. S1).

We used SEC coupled with multiangle light scattering (SEC-MALS) to further analyze the effects of pH and NaCl concentration on IDOL homogeneity (Fig. S1). This revealed that IDOL runs as a dimer or larger oligomers, with peak widths that suggest polydispersity. Among the different conditions tested, its monodispersity is optimal in a buffer containing 20 mm MES, pH 6, and 500 mm NaCl. Because monodispersity can promote crystallization (29), we decided to change the IDOL purification protocol by replacing Tris buffer, pH 8, with MES buffer, pH 6, for all the purification steps and using 20 mm MES, pH 6, 500 mm NaCl, 1 mm TCEP as storage buffer.

### In vitro IDOL E3 ligase activity

Using these optimized conditions for purification, we tested the ability of IDOL to promote polyubiquitin chain formation *in vitro*. Similar to what has been reported for the IDOL RING domain alone (14, 17), full-length IDOL promotes robust autoubiquitination *in vitro*. Although both E2s tested supported IDOL-mediated ubiquitination, activity is more pronounced for UBCH5C (UBE2D3) (Fig. 1*C*).

We next evaluated the activity of full-length IDOL protein against its substrate, the LDLR, using a fluorescently labeled LDLR peptide containing the IDOL recognition and ubiquitination sites (LDLR residues 811–835). We observed IDOL-dependent ubiquitin modification of the LDLR peptide, and this activity was not present on a random lysine-containing peptide or with an alternative E3 ligase, RNF168 (Fig. 1*D*). In contrast to efficient IDOL-stimulated ubiquitination of the LDLR in mammalian cells (12, 14, 17), the reaction did not seem very efficient, suggesting that IDOL is not fully active in modifying this peptide substrate or that a cell-specific activation signal is missing (Fig. 1*D*). Additionally, despite extensive attempts, no significant binding interaction could be measured with a variety of LDLR-derived peptides in fluorescence polarization or surface plasmon resonance experiments (data not shown), further supporting the notion that additional interactions, conformational changes, or post-translational modifications may be required for efficient ubiquitination.

### FERM domain crystal structures

To investigate the mechanism underlying the IDOL-LDLR interaction, we therefore proceeded with structural studies. Unfortunately, neither the original purified full-length IDOL nor the protein purified under the optimized buffer conditions crystallized to allow structural determination of the full-length IDOL protein. Therefore, we purified a series of IDOL FERM domain truncations for crystallization (Fig. 1*A*). The constructs were eluted as symmetric peaks from SEC columns in purification and showed well-defined melting transitions, indicating well-folded proteins (Fig. S3). We obtained diffracting crystals for two different constructs: IDOL FERM domain, which spans residues 1–344 (FERM^344^), and the third lobe of the FERM domain, F3ab (residues 183–283).

The F3ab subdomain crystallized in space group P2_1_2_1_2_1_, with cell dimensions *a* = 56.37 Å, *b* = 69.54 Å, *c* = 74.32 Å, α = β = γ = 90°, with three subunits in the asymmetric unit and a solvent content of 43%. We collected a single crystal data set, which diffracted to 2.35 Å resolution and solved F3ab crystal structure by molecular replacement using the equivalent region from DAL-1 (PDB ID: 2HE7) as a search model. Alternate model building in *Coot* (30) and refinement in BUSTER (31) resulted in a structure with *R*/*R*_free_ of 25.4/28.4 and overall good statistics (Table 1).

**Table 1:** **X-ray data collection and refinement statistics** Values for the highest-resolution shell are shown in parentheses. RMSD, root mean square deviation.

Crystal	FERM^344^	F3ab
**Data collection**		
Beamline	ESRF MASSIF-1	ESRF ID23-1
Wavelength (Å)	0.966	0.979
Resolution range (Å)	56–2.40	74–2.34
Space group	I4_1_22	P2_1_2_1_2_1_
Cell parameters		
*a*, *b*, *c* (Å)	159.49, 159.49, 76.72	56.37, 69.54, 74.32
α, β, γ (degrees)	90, 90, 90	90, 90, 90
No. of unique reflections	19,675	12,704
Completeness (%)	99.8 (97.7)	99.3 (97.2)
Redundancy	7.1 (6.7)	4.6 (4.4)
*I*/σ(*I*)	12.7 (0.2)	11.0 (0.7)
**Refinement statistics**		
Resolution range (Å)	50–2.5	50–2.34
*R*-factor/*R*_free_ (%)	22.9/26.4	25.4/28.4
Reflections (working/free)	17,399/841	12,627/619
Molecules per asymmetric unit	1	3
RMSD from target		
Bond lengths (Å)	0.009	0.008
Bond angles (degrees)	1.08	1.04
Ramachandran plot		
Favored (%)	94.8	94.6
Allowed (%)	5.2	5.4
Outlier (%)	0	0

The FERM^344^ protein crystallized in space group I4_1_22, with unit-cell parameters *a* = *b* = 159.49 Å, *c* = 76.72 Å, α = β = γ = 90°, with one molecule in the asymmetric unit and a solvent content of 60%. We collected a 2.5 Å data set and solved its crystal structure by molecular replacement using the primitive monoclinic crystal structure of the FERM domain of protein 4.1R (PDB ID: 3QIJ) as search model. After several cycles of alternate model building in *Coot* and structure refinement in BUSTER and Refmac (32), we achieved a structure with *R*/*R*_free_ of 22.9/26.4% and overall good statistics (see Table 1). The 301–305 loop could not be resolved in density as well as the last 12 C-terminal residues. In addition, the loops in the F2 region are less well-defined, most likely due to flexibility.

**Figure 2.**
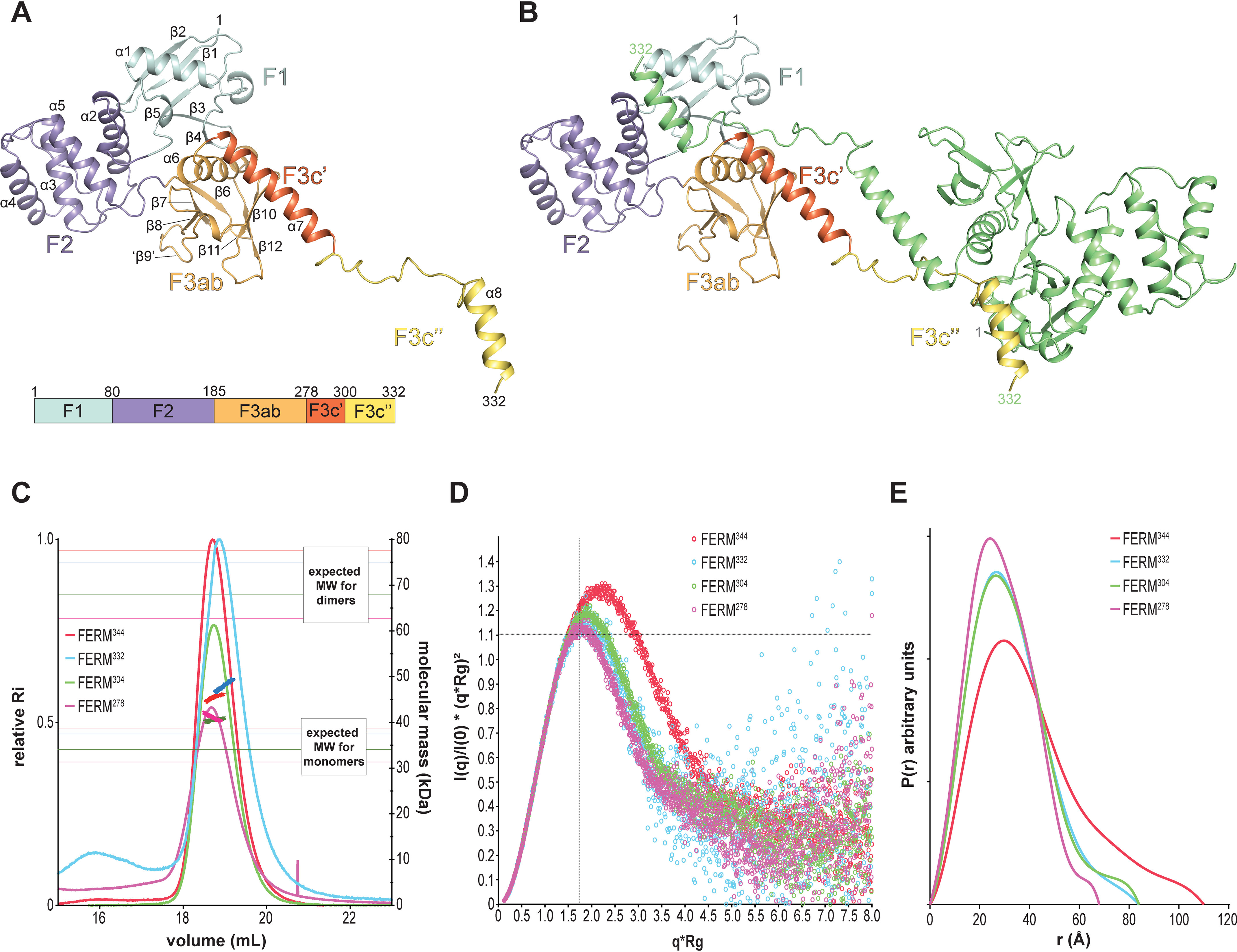
**Crystal structure of FERM^344^.** *A*, *cartoon representation* of the FERM^344^ crystal structure. The *color scheme* is as in Fig. 1*A*. The N- and C-terminal residues and subdomain boundaries are indicated. Strand “β9” has lost its secondary structure propensity in FERM^344^, whereas its structure is maintained in F3ab structure (see also Fig. S4). *B*, crystal contact interface between FERM^344^ and symmetry-mate subunit (*colored* in *light green*, generated by symmetry operation −*x*−1,−*y*,*z*). *C*, SEC-MALS analysis of the oligomeric states of FERM variants. Refractive index profiles for all variants are shown in *thin lines*, whereas the *thick lines* (at peak position) represent the MW distribution (g/mol) of the corresponding variant. Theoretical MW of the monomeric form for each variant is reported. *D*, SAXS shape analysis. Comparison of dimensionless Kratky plots of FERM variants. FERM^278^ shows the most compact conformation with the relative Kratky plot perfectly centered at √3. FERM^304^ and FERM^332^ show a very similar Kratky profile, with the maxima slightly shifted but still quite compact. FERM^344^ shows the less compact conformation among the variants. *R_g_* values calculated by the Guinier approximation for all the samples are listed in Table 2. *E*, SAXS pair-distribution function *P*(*r*) of FERM variants. FERM^278^ is characterized by a unimodal distribution, indicative of a globular conformation. FERM^304^ and FERM^332^ have a similar *D*_max_, which indicates that, despite the flexibility of F3c″ domain in solution, it folds back onto the same molecule at its C terminus and that a subpopulation of extended conformations exists in solution. *D*_max_ and *R_g_* values obtained from the analysis of the *P*(*r*) function are listed in Table 2.

The F1, F2, and F3 regions of FERM^344^ adopt the archetypical cloverleaf structure of the FERM domains (Fig. 2*A*). Previous sequence analysis (28) had suggested that IDOL FERM domain could contain an apparent insertion within the F3 subdomain. However, our structure shows that F3ab runs from 186 to 278 and that the region spanning residues 279–332 is clearly outside the cloverleaf region. Based on the obtained structure, we propose to redefine IDOL FERM boundaries as shown in Fig. 1*A*, with the extra region in FERM (which spans residues 279–344) divided into three subdomains to accentuate the extension away from the F3ab lobe: F3c′ (residues 279–300), F3c″ (residues 301–332), and F3c‴ (residues 333–344), with the latter not traceable in the electron density map (Fig. 2*A*).

The FERM^344^ subdomain F1 spans residues 1–80 and adopts a ubiquitin-like fold, consisting of a five-stranded β-sheet, a short 3_10_ helix, and an α helix. Strands β1 and β5 are the first and the last β strand in the F1 subdomain, respectively, but form consecutive strands in the F1 β-sheet, resulting in an overall compact F1 domain. Subdomain F2 spans residues 81–185 and is all helical. It is composed of a four-helix bundle characteristic of the acyl-CoA–binding protein fold. The last subdomain in the traditional FERM cloverleaf is subdomain F3ab, encompassing residues 186–278. This domain resembles a phosphotyrosine-binding domain, characterized by the pleckstrin homology superfold, with seven antiparallel β-strands forming two orthogonal β-sheets capped by a C-terminal α helix (α6, residues 258–276). After the α7 helix, the FERM^344^ structure has a partially unstructured region from residue 301 until residue 317, followed by helix α8 (residues 318–332) (F3c″), which is the last residue traceable in the electron density map.

The F3ab conformation is well-conserved between the FERM^344^ and the three copies in the asymmetric unit of the F3ab crystal structure. There is a conformational difference in two β-turns (β6-β7 and β8-β9) in the FERM^344^ structure due to crystal contacts, which results in a loss of β-sheet character for strand “β9” in this structure (Fig. S4). Moreover, there are clear differences in the F3c′ region, where the FERM^344^ crystal structure has an α helix (α7), following the F3ab, which spans residues 279–300 and folds back onto the F3ab. This region has lost helical conformation in all three copies of the F3ab structure, and in one case (F3ab monomer B) even the previous helix α6 is partially unraveled (Fig. S4).

### FERM^344^ oligomerization state

The FERM^344^ domain packs as a tetramer in the crystal created by crystallographic symmetry operations (Fig. S5). Its extended F3c′-F3c″ region (residues 279–332) interacts extensively with F1 and F3 of a symmetry-related molecule, burying a substantial surface area of 2537 Å^2^ (Fig. 2*B*). This dimer has smaller crystallographic interfaces to another identical dimer (840 and 273 Å^2^, respectively), generating a tetramer, with a central four-helical bundle formed by helix α7 (F3c′ subdomain) (Fig. S5).

Such packing and especially the large dimer interface could indicate that oligomerization is important for IDOL function. Therefore, we investigated the solution behavior of different IDOL constructs, using SEC-MALS. As shown in Fig. 2*C*, all the FERM variants presented in this study behave as monomers, indicating that the oligomerization is only relevant under crystallization conditions. The monomeric state of FERM^344^ was also confirmed by small-angle X-ray scattering (SAXS) analysis (see below) (Table 2 and Fig. 2 (*D* and *E*)).

**Table 2:** **SAXS-derived structural parameters and fitting statistics**

	SAXS experimental scattering data			
	FERM^278^	FERM^304^	FERM^332^	FERM^344^
**SAXS-derived parameters**				
Guinier				
*R_g_* (Å)	22.32	25.17	24.95	32.27
*P*(*r*) function				
*R_g_* (Å)	22.32 ± 0.04	25.17 ± 0.04	24.96 ± 0.09	32.30 ± 0.06
*D*_max_ (Å)	68	84	84	110
MW estimation				
*Q_P_* (kDa)*^a^*	26.2	35.4	33.4	54.3
MoW (kDa)*^a^*	32.0	39.2	38.9	59.9
*V*_c_ (kDa)*^a^*	29.2	34.9	34.6	50.9
Size and shape (kDa)*^a^*	30.3	37.9	34.6	60.2
Bayesian inference (kDa)*^a^*	28.9	36.9	34.6	55.6
MW based on composition				
Calculated MW (kDa)*^b^*	31.2	34.1	37.5	38.8
**Calculated scattering data**				
With CRYSOL				
FERM^278^	1.09	4.14	1.23	36.13
FERM^304^	0.97	2.83	1.11	22.92
FERM^332^	5.95	3.02	1.38	5.89
With SREFLEX				
FERM^304^		1.02	1.01	5.84
FERM^332^			1.05	1.58

*^a^*Derived from five independent methods (33), as implemented in PRIMUS (34). *Q_p_*, Porod invariant; MoW, molecular weight; *V*_c_, volume of correlation.

*^b^*Calculated from primary sequence of individual truncations with the ProtParam server (RRID:SCR_018087) (35).

### Comparison with other FERM domains

Having obtained the IDOL FERM domain structure, we proceeded to compare it with other FERM domains in the PDB.

**Figure 3.**
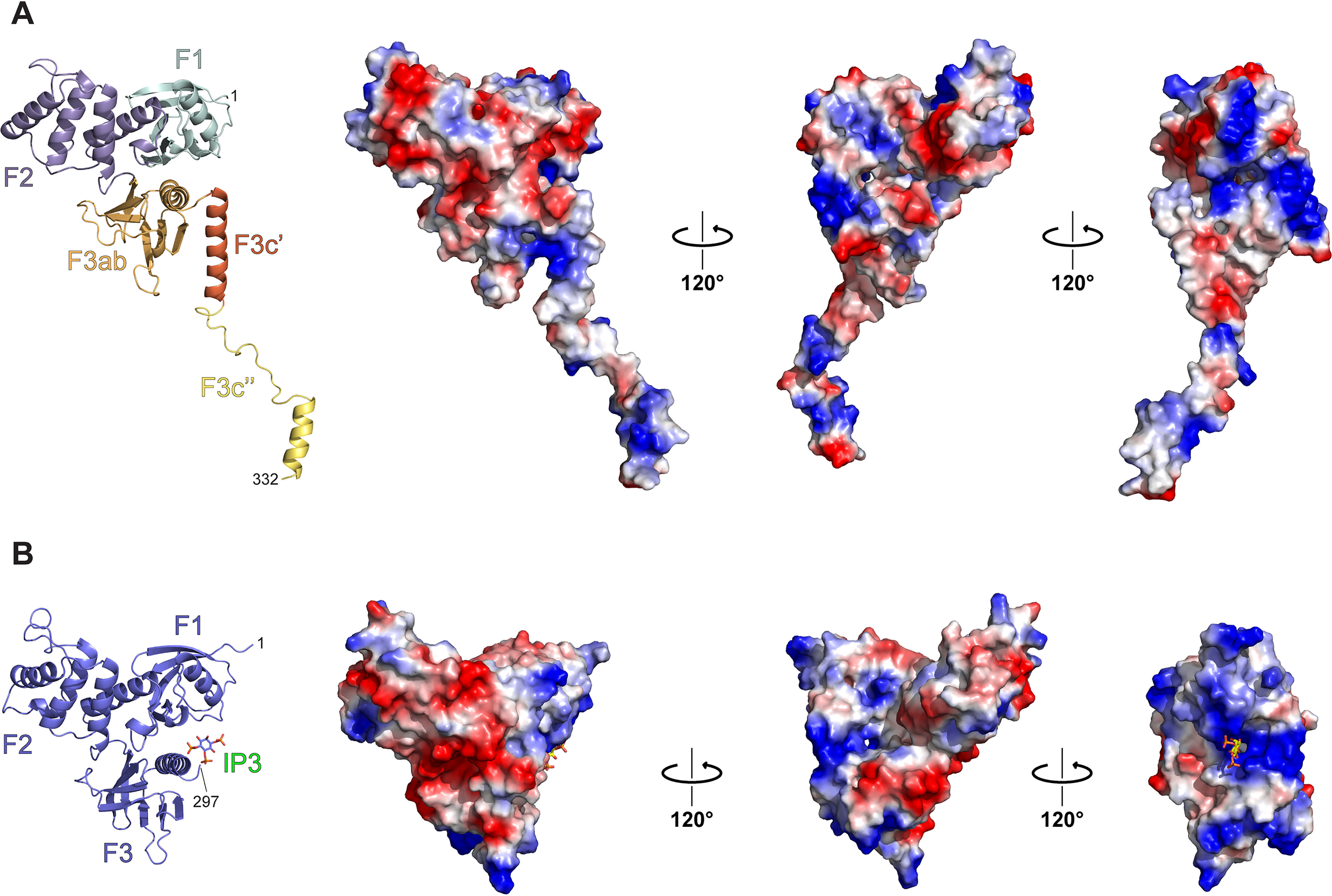
**Surface charge distribution of FERM^344^.** Shown is a comparison of electrostatic surface potentials of FERM^344^ (*A*) and human radixin (PDB ID: 1GC6) (*B*). The electrostatic surface potentials are represented *over* the *protein surface* in *blue* and *red shades* for positively and negatively charged residues, respectively. Although positive charges are somewhat conserved around the radixin IP_3_-binding site (represented in a *stick model*), the three interacting lysine residues are not conserved in IDOL.

First, we analyzed the surface charge properties of the IDOL FERM domain (Fig. 3*A*). Some FERM domains, such as radixin, have an inositol trisphosphate (IP_3_)-binding site between the F1 and F3ab subdomains. Analysis of the surface properties of the IDOL FERM domain shows that the general positive character of this surface is somewhat conserved (Fig. 3*B*), but not all coordinating residues are. There are three lysines (Lys^60^, Lys^63^, and Lys^278^) involved in the IP_3_ binding in radixin (PDB ID: 1GC6) (36), whereas in IDOL, those residues are replaced with two arginines (Arg^59^ and Arg^61^) and one serine (Ser^262^), respectively, with a very different arrangement.

**Figure 4.**
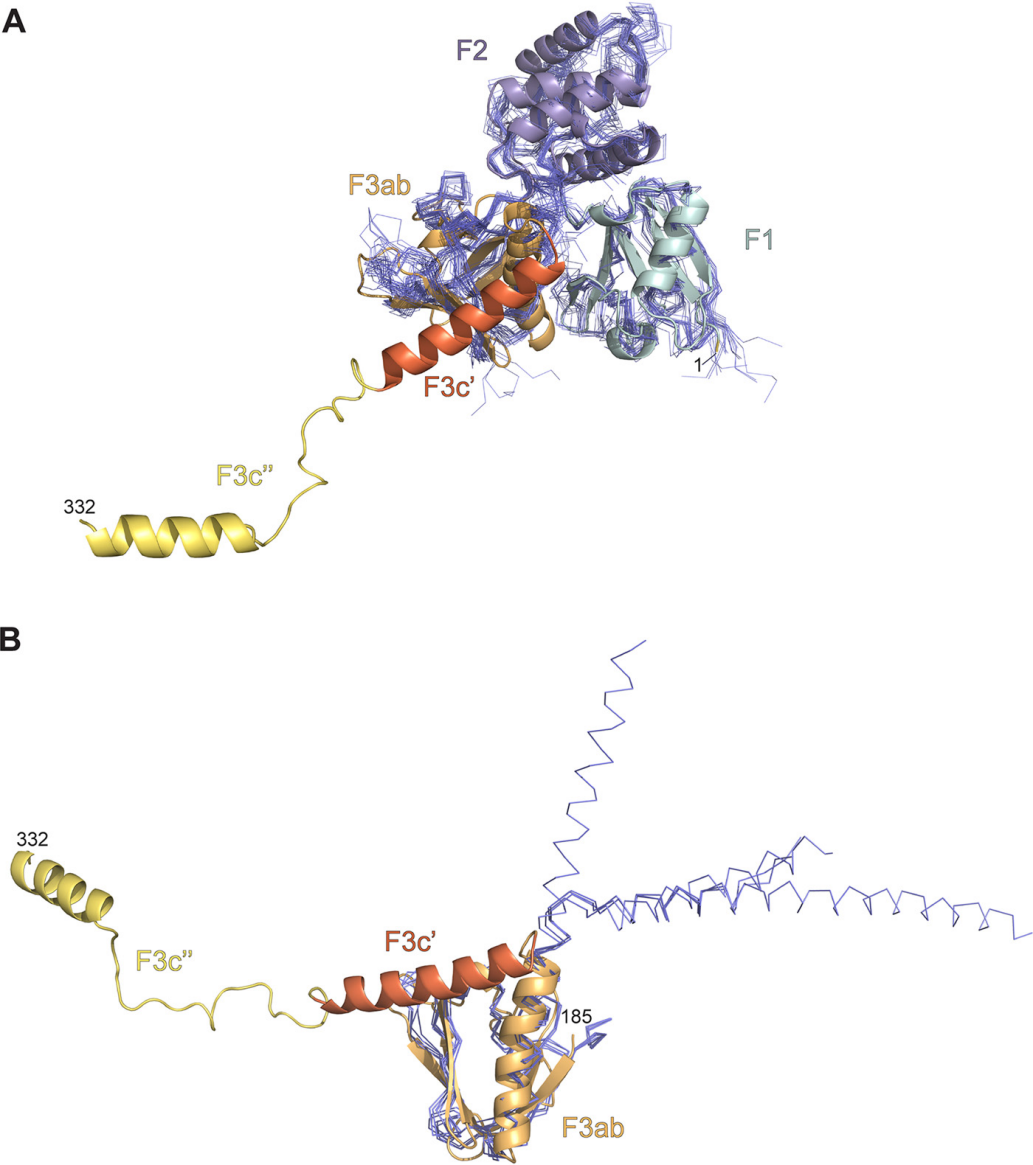
**FERM^344^ subdomain structural comparisons.** *A*, overall structural comparison of FERM^344^ against other FERM members. *B*, F3c′ differences in other FERM domain proteins.

Using SSM (37), we performed an overall structural alignment to FERM domain structures where the full cloverleaf structure was available (Fig. 4*A*). Superposition of 27 structures shows that the characteristic cloverleaf structure is well-conserved in FERM^344^ and that a helix similar to the F3c′ region is also observed in human moesin (PDB ID: 1E5W) (38), mouse merlin (PDB ID: 1ISN) (39), human merlin (PDB ID: 6CDS) (40), and mouse radixin (PDB ID: 1J19 and 2EMS) (41, 42). Small variations in relative positioning and orientation of the F1, F2, and F3ab domain were visible, but we observed similar variations for these subdomains between data sets from different FERM^344^ crystals. A major difference in other FERM domains is the position of the F3c′ region. This is differently oriented in the IDOL FERM domain (Fig. 4*B*), and we could not identify an equivalent of the F3c″ subdomain in other FERM-containing proteins.

### Conformation of IDOL FERM in solution

We wondered whether this unusual conformation of F3c relative to the FERM domain is relevant in solution. We used SAXS to characterize a series of FERM domain variants (see Fig. 1*A*) to assess the orientation of F3c′ and the extended conformation of F3c″ separately.

After checking that the scattering profiles of the FERM variants did not show any sign of possible aggregation, we performed Guinier fitting on the SAXS data to estimate the radius of gyration (*R_g_*) (Table 2). Next, we generated dimensionless (normalized) Kratky plots (43) to assess the degree of compactness of the FERM truncations in solution. The dimensionless Kratky plots for all the FERM variants have a characteristic bell shape, indicative of folded and compact structures (Fig. 2*D*). However, only the FERM^278^ variant has its peak centered closed to √3, indicative of a particularly compact structure, whereas all the other constructs have their maxima shifted to higher values of *q* × *R_g_* (around 1.85 for FERM^304^ and FERM^332^ and around 2.25 for FERM^344^), suggesting properly folded but more elongated conformations (Table 2).

To gain more insight into the shape and the size of these FERM variants, we analyzed the pair distribution function, *P*(*r*), a weighted histogram of all possible pair distances between pairs of atoms within a particle (Fig. 2*E*). By extrapolating the *r* values at which *P*(*r*) goes to zero, this analysis estimates the maximum dimension (*D*_max_) of the biomolecule in solution. FERM^304^, FERM^332^, and FERM^344^ *P*(*r*) functions have a similar profile, with a noticeable tail, short for FERM^304^ and FERM^332^ and somewhat longer for FERM^344^. This suggests that the F3c′-F3c″-F3c‴ region extends away from the body of the FERM F1-F2-F3ab and is characterized by a certain degree of flexibility. *D*_max_ values derived from the *P*(*r*) function for FERM^278^ (68 Å) and FERM^304^ (84 Å) are in very good agreement with their maximum dimensions calculated from the FERM^344^ crystal structure, suggesting that the F3c′ helix closely interacts with the F1-F2-F3ab core also in solution. The *D*_max_ for FERM^332^ (84 Å) is not larger than that of FERM^304^, indicating that in solution, the F3c″ domain folds back onto the F1-F2-F3ab globular core instead of adopting the extended conformation seen in FERM^344^ crystal structure. The extended *D*_max_ for FERM^344^ (110 Å) shows that the last 12 C-terminal residues are highly flexible. We compared the SAXS data with calculated scattering profiles, computed from our crystal structure of the FERM domain, including only the relevant residues of FERM^278^, FERM^304^, and FERM^332^. The calculated data are in good agreement with the corresponding experimental scattering data, with FERM^304^ having the worst fit (χ^2^ = 2.83; see Table 2). Interestingly, experimental scattering data collected for FERM^344^ does not fit with the theoretical scattering profiles calculated with CRYSOL for either FERM^278^-, FERM^304^-, or FERM^332^-derived structures (see Table 2). However, if we allow flexibility and conformational changes of subdomains F3c′ and F3c″ with SREFLEX, the obtained FERM^304^ and FERM^332^ “flexible” models better resemble the experimental scattering data (see Table 2), supporting an intrinsic flexibility of subdomains F3c′, F3c″, and F3c‴. Interestingly, this keeps the F3c′ bent toward the F3ab subdomain.

In conclusion, our SAXS data confirm the observation that the F3c′ helix is predominantly bent back toward the bulk of the F3ab domain, whereas the F3c″ and F3c‴ are most likely flexible. These data suggest that the unusual orientation of the F3c′ helix observed in the crystal structure is also valid in solution.

### Analysis of the LDLR peptide-binding site

**Figure 5.**
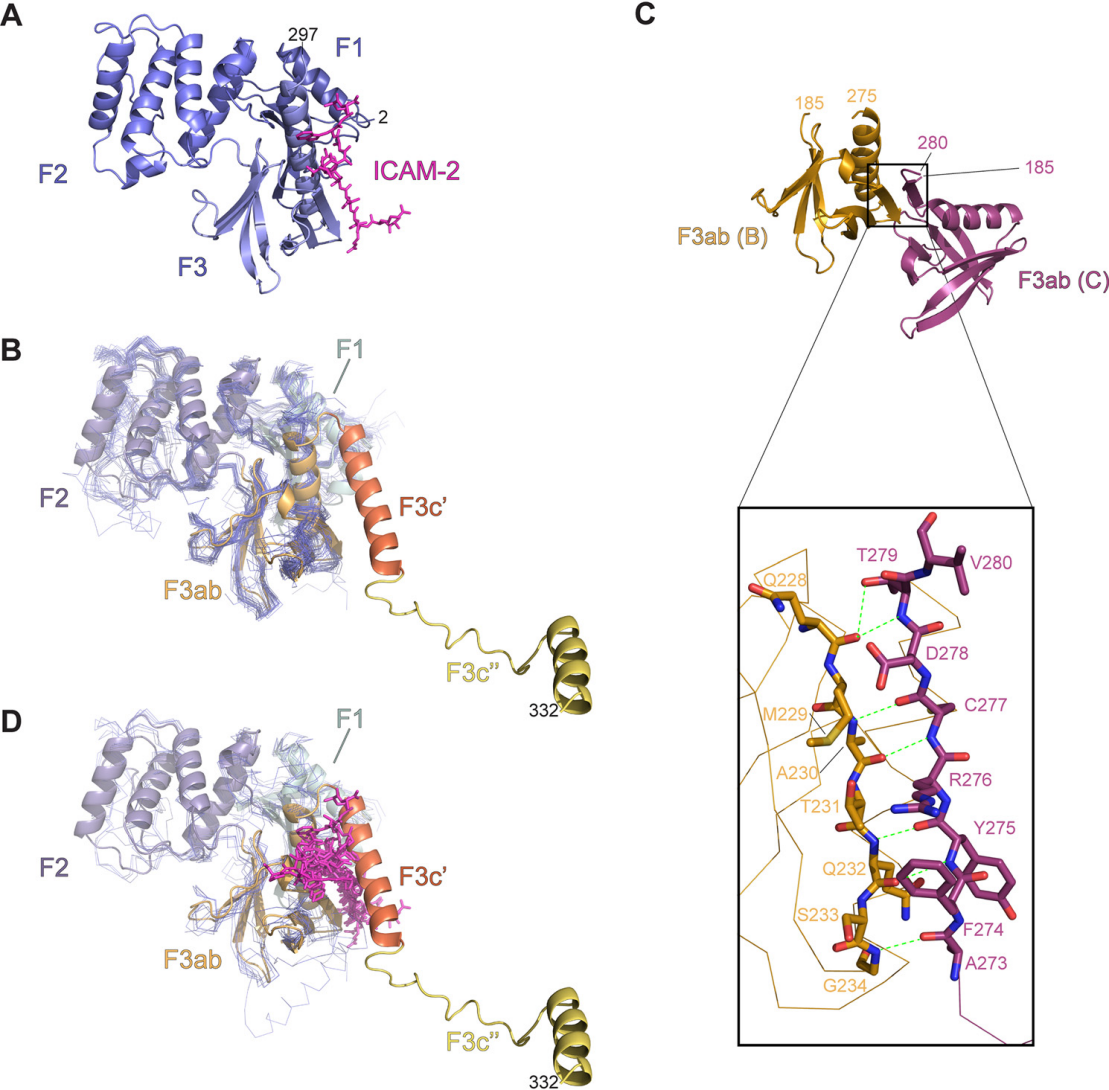
**FERM^344^ peptide-binding region.** *A*, RADIXIN 1J19 FERM domain (in a *cartoon representation*, *light blue*) in complex with intercellular adhesion molecule 2 (*ICAM-2*) cytoplasmic peptide (in a *stick representation*, *magenta*). *B*, superposition of FERM domains (*blue*) on FERM^344^ (*colors* according to Fig. 1*A*) showing how in IDOL the F3c′ subdomain closes back onto the F3ab subdomain. *C*, *ribbon representation* of F3ab crystal structure. The *color scheme* is as in Fig. 1*A*. Monomer B and monomer C, with respective N- and C-terminal residue boundaries are indicated. *Inset*, interaction between F3ab monomer B and the C-terminal tail of F3ab monomer C, with corresponding hydrogen bonds indicated. *D*, structural superposition of FERM^344^ and FERM domain proteins complexed with their respective peptide ligands (PDB IDs used for the structural comparison: 1NTV, 1J19, 2EMS, 2ZPY, 3BIN, 4GXB, and 4TKN).

FERM domains are important mediators of protein-protein and protein-membrane interactions. An important conserved region in this context is the peptide interaction site within the F3ab domain. Several structures have been determined to delineate how FERM domains interact with peptides of their cognate partners at this site. For IDOL, the interaction with the LDL receptor has been mapped to this location, with residues Tyr^265^, Thr^269^, and His^272^ being important for E3 ligase activity on the LDL receptor in cells (14, 28). The interaction in related FERM domains consists of a main-chain peptide interaction (Fig. 5, *A* and *B*) (41, 42, 44–47), forming an extra β-strand in the F3ab sheet, with hydrophobic side chains giving sequence specificity. Upon superposition of the FERM domain structure, the mutants most critical for binding are indeed found to line the peptide-binding groove (14, 28).

### The F3c′ helix obscures the LDLR peptide-binding site

In the FERM^344^ structure, the F3c′ is helical and positioned over the F3ab domain, obscuring the access to the peptide binding site. If this particular position is retained in full-length IDOL, it could prevent the interaction with the LDLR intracellular tail, suggesting a possible form of autoinhibition. Because our SAXS data suggest that this conformation is maintained in solution, it is possible that this is indeed the case. This could potentially explain the low affinity and activity observed for full-length IDOL against the LDL receptor peptide in our *in vitro* binding and ubiquitination assays. Because the expression construct used to crystallize F3ab has an incomplete F3c′, helix α7 is unfolded and helix α6 is shorter in all of the three F3ab monomer structures. In fact, in two of the three copies in the asymmetric unit, this unraveled region contacts another F3ab molecule (Fig. S4), where it takes on the role of the interacting peptide. The unraveled peptide binds in the F3ab peptide-binding site, making an interaction that closely resembles the β-β association observed in many FERM-substrate peptide complexes (Fig. 5, *C* and *D*). This confirms that the F3ab subunit is intrinsically capable of forming the peptide interaction observed in other FERM receptor interactions. However, in the FERM^344^ structure, the α7 helix obscures access.

The general positive character of IDOL FERM surface may be important for membrane recognition and IDOL activity (Fig. 3). Three IDOL residues (Arg^193^, Lys^199^, and Arg^259^) were shown to be necessary, *in vitro*, for the recognition and interaction of IDOL with negatively charged phospholipid vesicles (28). Similarly, a R193E/K199E/R259E IDOL mutant construct showed reduced degradation of the LDLR, supporting the idea that the membrane interaction is a key component in the IDOL-LDLR interaction (28). These three residues are indeed found on the surface, in a positively charged surface patch that also includes residues Lys^50^, Lys^235^, and Lys^254^.

It will be interesting to see whether the orieniation of the F3c′ helix is relevant in full-length IDOL. If so, this could possibly be a novel form of FERM domain autoinhibition, explaining the low ubiquitination activity of IDOL on an LDLR peptide. It is possible that the conformation of the LDLR tail in a more physiological setting (*e.g.* as part of a transmembrane protein) is critical. Alternatively, post-translational modifications of IDOL may be required for optimal recognition of the LDLR, or some form of membrane association could trigger the necessary conformational change to unmask the peptide-binding site. Ultimately, it will be interesting to see whether IDOL autoinhibition resembles the autoinhibition observed in other FERM domain proteins or if it expands the mechanisms employed for this purpose.

## Conclusions

Here we present two crystal structures of the FERM domain of human IDOL. These structures show that although IDOL has the canonical cloverleaf structure found in FERM domains, its F3c′ adopts a conformation that may obscure substrate access to its F3ab domain. This may suggest that an additional signal is required for full activation of IDOL activity.

## Experimental procedures

### Cloning

Full-length human IDOL (residues 1–445) was cloned with a TEV-cleavable N-terminal His_6_ tag into pFastbac1 for baculovirus expression. Recombinant baculovirus was generated using the Bac-to-Bac system. For the generation of FERM variants, full-length IDOL (residues 1–445) was cloned into the pET-NKI-hisSUMO2 expression vector of the NKI LIC (ligation-independent cloning) suite (48). From this pET-NKI-hisSUMO2-IDOL template, FERM variants were generated by C-terminal truncation through introduction of a premature stop codon by QuikChange site-directed mutagenesis (Agilent) immediately after IDOL residue 278 (referred to here as FERM^278^), residue 304 (FERM^304^), residue 332 (FERM^332^), and residue 344 (FERM^344^). The *E. coli* codon–optimized gene of the human F3ab subdomain (residues 183–283) with an N-terminal TEV-cleavable His_6_ tag was purchased from GenScript and cloned into a pET28(a) vector using NcoI and XhoI restriction enzyme sites. The insect cell (full-length IDOL) and bacterial (FERM variants and F3ab subdomain) expression constructs used in this work are summarized in Fig. 1*A*.

### Protein expression and purification

For full-length IDOL, *Spodoptera frugiperda* Sf21 cells cultured in Sf900II SFM medium at 27 °C were infected with baculovirus (multiplicity of infection = 2) at a cell density of 2.5–3.0 × 10^6^ cells ml^−1^ and harvested after 72 h. Cell pellets were resuspended in 50 mm Tris, pH 8.0, 500 mm NaCl, 1 mm TCEP, 15 mm imidazole, Benzonase, and 1 tablet of cOmplete™ protease inhibitor mixture (Sigma–Aldrich) per 50 ml of lysis buffer and lysed by sonication for 2.5 min on ice. The lysate was than centrifuged (55,000 × *g* for 1 h at 4 °C) and loaded on a 5-ml HisTrap HP column (GE Healthcare). The resin was washed with lysis buffer supplemented with buffered 25 mm imidazole, and the protein was eluted with lysis buffer supplemented with 500 mm imidazole at pH 8. The elution fractions were pooled; diluted 1:3 with 20 mm Tris, pH 8.0, 1 mm TCEP; immediately loaded on an anion-exchange column (POROS XQ, Thermo Fisher Scientific); and eluted with a linear NaCl gradient. IDOL fractions were concentrated and loaded on a size-exclusion chromatography column (Superdex S200, GE Healthcare) previously equilibrated with 20 mm Tris, pH 8, 200 mm NaCl, 1 mm TCEP. Peak fractions were concentrated to about 6 mg ml^−1^, aliquoted, and snap-frozen in liquid nitrogen for storage.

All FERM variant plasmids were transformed into Rosetta2 (DE3) T1^R^ *E. coli* cells. Cells were grown in TB medium, in the presence of kanamycin and chloramphenicol, at 37 °C while shaking until the *A*_600_ was 1.2–1.5. Cultures were then induced with 0.5 mm isopropyl 1-thio-β-d-galactopyranoside and allowed to grow overnight at 18 °C. Cells were harvested by centrifugation (5000 × *g* for 20 min at 4 °C) and resuspended in lysis buffer containing 50 mm Tris, pH 8, 500 mm NaCl, 1 mm TCEP, 25 mm imidazole, DNase, and one tablet of cOmplete™ protease inhibitor mixture (Sigma–Aldrich) per 50 ml of lysis buffer. The purification protocol for all the FERM variants was derived from Ref. 49. Briefly, clear lysate was loaded on nickel affinity resin beads previously equilibrated with 50 mm Tris, pH 8.0, 500 mm NaCl, 1 mm TCEP, 15 mm imidazole. The resin was then washed with lysis buffer supplemented with 25 mm imidazole (pH 8), and the protein was eluted with lysis buffer supplemented with 500 mm imidazole (pH 8). SUMO tag was removed by pooling the elution fractions and incubating with SENP2 protease for 45–60 min at room temperature while dialyzing against 20 mm Tris, pH 8.0, 150 mm NaCl, 1 mm TCEP, followed by a reverse affinity chromatography step. Once cleaved, FERM variant proteins were individually buffer-exchanged to 20 mm MES, pH 6.8, 150 mm NaCl, 1 mm TCEP and immediately loaded on a cation-exchange column (POROS XS, Thermo Fisher Scientific) and eluted with a linear NaCl gradient. FERM fractions were then concentrated and loaded on a size-exclusion chromatography column (Superdex S200, GE Healthcare) previously equilibrated with 20 mm MES, pH 6.8, 300 mm NaCl, 1 mm TCEP. Peak fractions were concentrated to about 6–10 mg ml^−1^, snap-frozen in liquid nitrogen, and aliquoted for storage.

FERM F3ab pET28(a) plasmid was transformed in BL21(DE3) *E. coli* cells. Cells were grown in lysogeny broth medium, in presence of kanamycin, at 37 °C while shaking until the *A*_600_ was 0.6–0.9. Cultures were induced with 0.1 mm isopropyl 1-thio-β-d-galactopyranoside and allowed to grow overnight at 18 °C while shaking. Cells were harvested by centrifugation and resuspended in lysis buffer containing 50 mm Tris, pH 8, 300 mm NaCl, 10% glycerol, 1 mm TCEP, 25 mm imidazole supplemented with Benzonase and one tablet of cOmplete™ protease inhibitor mixture and lysed by cell disruption. The lysate was than centrifuged and loaded on a 5-ml HisTrap HP column (GE Healthcare). The column was washed with lysis buffer, and the protein was eluted with lysis buffer supplemented with 350 mm imidazole at pH 8. The eluted protein was then incubated with TEV protease overnight at 4 °C while dialyzing against 50 mm Tris, pH 8, 300 mm NaCl, 10% glycerol, 1 mm TCEP, 25 mm imidazole, followed by a reverse affinity chromatography step. Cleaved F3ab protein was concentrated and loaded on a size-exclusion chromatography column (Superdex S75, GE Healthcare) previously equilibrated with 50 mm MES, pH 6.5, 500 mm NaCl, 1 mm TCEP, 10% (v/v) glycerol. Peak fractions were concentrated to about 6 mg ml^−1^, snap-frozen in liquid nitrogen, and aliquoted for storage. In all cases, after the size-exclusion chromatographic step, the purity and homogeneity of each protein was assessed by SDS-PAGE.

### Differential scanning fluorimetry for full-length IDOL

Thermal unfolding profiles of full-length IDOL (25 μl at 1 mg/ml) were recorded, in the presence of different buffer and salt compositions using DSF in a MyiQ RT-PCR system (Bio-Rad) by monitoring the fluorescent intensity of SYPRO orange (excitation, 485 nm; emission, 575 nm) with a temperature gradient of 0.5 °C/min between 15 and 95 °C and a dwell time of 10 s/step.

### Measurements of apparent T_m_ for FERM truncations

Temperature-dependent conformational changes were measured using nano-DSF in a Prometheus NT.48 (NanoTemper Technologies). Each FERM variant was diluted in 20 mm MES, pH 6.8, 300 mm NaCl, 1 mm TCEP to the desired concentrations. Measurements were taken over a temperature gradient of 1 °C/min between 20 and 95 °C, using an excitation power of 10%. The measured 350 nm/330 nm intensity ratios were plotted *versus* temperature. The *T_m_* values were determined from the maximum of the first derivatives of these spectra using Prometheus NT.48 internal software.

### SEC-MALS

SEC-MALS measurements were performed on an ÄKTApurifier 100 (GE Healthcare) connected to a tri-angle detector MiniDAWN Tristar (Wyatt Technologies). For each experiment, a volume of 100 μl of 100 μm protein solution was injected into a Superdex 200 10/300 column (GE Healthcare), previously equilibrated with 20 mm MES, pH 6.8, 300 mm NaCl, and 1 mm TCEP. Molecular weights of main peaks were determined using the manufacturer's software (ASTRA) and assuming a specific refractive index increment (d*n*/d*c*) of 0.185 ml g^−1^. Chromatographic profiles and molecular weights were plotted using Prism 7 (GraphPad).

### Immunoblotting

SDS gels used were all precast 4–12, 10, or 12% BisTris gels (Thermo Fisher Scientific) run in MES or MOPS buffer (Thermo Fisher Scientific) and blotted on polyvinylidene difluoride membrane (pore size 0.45 μm; Sigma–Aldrich) with standard blotting buffer. Blocking of the Western blots was done in 5% skim milk (Merck Millipore) in PBS-Tween 20. Antibodies used for Western blotting analysis were as follows: for ubiquitin, P4D1 (horseradish peroxidase–conjugated mouse mAb from Santa Cruz Biotechnology, catalog no. sc-8017; 1:5000 dilution), MYLIP/IDOL (goat polyclonal antibody from Everest Biotech, catalog no. EB09591; 1:5000 dilution). The secondary antibody used was swine anti-goat horseradish peroxidase–conjugated from BioSource International, catalog no. ACI3404, 1:1000–2000 dilution.

### In vitro ubiquitination assays

Recombinant human UBA1, UBCH5C, RNF168, and ubiquitin were purified as reported previously (50). UBC13/UEV2 was a kind gift from Dr. Ben Distel (University of Amsterdam Medical Center). *In vitro* ubiquitination reactions were carried out at 37 °C for the time indicated for each experiment, in the presence of 25 mm Tris, pH 8, 150 mm NaCl, 3 mm MgCl_2_, 1 mm TCEP. Concentrations used were 5 mm ATP, 200 nm E1, 250 nm E2, 15 μm ubiquitin, 50 nm E3 ligase (IDOL or RNF168), and, if present, 15 μm ^TAMRA^LDLR_peptide (residues 811–835) or a 15 μm concentration of a random ^TAMRA^ctrl_peptide (GPLATSTPKNNG). Reactions were stopped by the addition of SDS-PAGE loading buffer and analyzed by immunoblotting.

### Crystallization

Purified FERM^344^ was concentrated using an Amicon Ultra centrifugal filter (Millipore) with a 10-kDa nominal molecular mass cutoff. Eventual dilutions were carried out using the SEC buffer (20 mm MES, pH 6.8, 300 mm NaCl, 1 mm TCEP). All the crystallization experiments were carried out using Mosquito (TTP Labtech) and 96-well two-drop MRC crystallization plates (Molecular Dimensions). Initial crystallization screenings identified few promising hits from the ComPAS suite (Qiagen) screen set up at 20 °C. After a few rounds of condition optimization, we were able to generate the crystal used in this study. Explicitly, the FERM^344^ crystal used to solve the structure was obtained with the sitting-drop vapor diffusion method at 4 °C, by mixing 100 nl of concentrated FERM^344^ protein at 5.8 mg ml^−1^ with 200 nl of reservoir solution containing 100 mm Tris, pH 8.5, 20 mm MgSO_4_, 4% (w/v) ethylene glycol, and 12% (w/v) 2-methyl-2,4-pentanediol. Except for this condition, FERM^344^ protein also crystallized in several other similar conditions, which all contained the following components: 100 mm Tris buffer, pH 8–8.5, 10-20 mm MgSO_4_ plus one or two alcohols at low percentage. Pyramidal-like crystals started appearing after 4–5 days and stopped growing after 2–3 weeks. Crystals were then cryoprotected via transfer into a drop of reservoir solution added with 20–25% (v/v) ethylene glycol. After incubation in the cryoprotectant solution, the crystals were looped and flash-cooled in liquid nitrogen before data collection.

The purified F3ab domain was concentrated to 5.3 mg ml^−1^ in a buffer containing 500 mm NaCl, 10% glycerol, 1 mm TCEP, and 50 mm MES, pH 6.5. Initial crystallization screening was done using the commercial Index (Hampton Research) and JCSG+ (Molecular Dimensions) screens with 150 + 150 nl vapor diffusion drops at 20 °C using a Mosquito LCP robot (TTP Labtech). Crystals from the most promising condition, containing 100 mm Hepes, pH 7.0, and 10% (w/v) PEG 6000, were flash-frozen in liquid nitrogen using 25% (v/v) glycerol as cryoprotectant and prescreened using an FR-E+ SuperBright rotating anode (Rigaku Corp.).

### Data collection, structure determination, and structure analysis

X-ray diffraction data for FERM^344^ were collected at a wavelength of 0.966 Å at 100 K at the beamline MASSIF-1 ID30A-1 (ESRF, Grenoble, France) (51, 52), equipped with a PILATUS3 2M detector (DECTRIS, Baden, Switzerland). Data corresponding to the crystal structure presented in this study was collected up to 2.4 Å from a single crystal and belonged to space group I4_1_22, with one copy per asymmetric unit. Data were indexed, integrated, and scaled with the XDS software package (53). For details of the diffraction data and the data-processing statistics see Table 1. Initial phases were obtained with Mr.BUMP (54), an automated scheme for molecular replacement (MR) in CCP4 7.0.060 (55), using 3QIJ as a search model. The initial MR solution obtained at 2.5 Å was then subjected to several cycles of manual model building in *Coot* (30) and TLS (translation/libration/screw) restrained refinement in Refmac (32) and BUSTER (31) (Global Phasing Ltd., Cambridge UK). The structure was eventually refined against native data to 2.5 Å resolution with final *R*_work_ = 22.9% and final *R*_free_ = 26.4% (Table 1). To avoid overfitting of the structures, the PDB_REDO server (https://xtal.nki.nl/PDB_REDO/) (56) was used to determine the best relative weight for the X-ray target function and the geometry or the *B*-factor restraints for the last cycle of refinement. The quality of the final model was validated using MolProbity (57) (Table 1). The PISA (Protein Interfaces, Surfaces, and Assemblies) (58) program available in the CCP4 suite was used to compute the interface areas between the FERM^344^ monomer present in the ASU and crystallographic neighbors and to predict the most stable form of multimer(s) in solution.

Full X-ray diffraction data for F3ab were collected at a wavelength of 0.979 Å at 100 K at beamline ID23-1 (ESRF, Grenoble, France), equipped with a PILATUS 6M detector (DECTRIS). Data corresponding to the F3ab crystal structure presented in this work were collected up to 2.5 Å resolution. The data were indexed and integrated with MOSFLM (59) and scaled with SCALA (60) in space group P2_1_2_1_2_1_, with cell dimensions of 56.4, 69.5, and 74.3 Å and three copies per asymmetric unit. Initial phases were obtained by MR in PHASER (61), using the equivalent Glu^183^–Ser^283^ fragment from DAL-1 (PDB ID: 2HE7) (44) as a search model. The F3ab structure was further refined by alternative cycles of model rebuilding in *Coot* and refinement in BUSTER (Global Phasing Ltd.). A final model composed of Gly^186^–Ala^283^ (chain A), Tyr^185^–Tyr^275^ (chain B), and Tyr^185^–Val^280^ (chain C) was refined to an *R*/*R*_free_ of 25.4/28.4%. Full data collection and refinement statistics can be found in Table 1. Model-derived *R_g_* and *D*_max_ for FERM^278^, FERM^304^, and FERM^332^ were calculated with MOLEMAN (62). FERM^278^ and FERM^304^ models were created by truncating the model to residue 278 and residue 304, respectively. Structural images have been prepared using PyMOL (PyMOL Molecular Graphics System, version 1.7.2.1 Schrödinger, LLC, New York). SSM (37) at the European Bioinformatics Institute (RRID:SCR_004727) and the RAPIDO web server (http://webapps.embl-hamburg.de/rapido/) (63) were used to structurally align the FERM^344^ PDB structure to typical members of FERM domain–containing proteins.

### SAXS data collection and analysis

SAXS diffraction data were collected at 12.1 keV at beamline B21 (Diamond Light Source), equipped with a PILATUS 2M detector (DECTRIS, Switzerland). Sample-to-detector distance was 4 m. Individual samples were injected at the following concentrations: FERM^278^ at 5.87 mg ml^−1^ (187 μm), FERM^304^ at 7.73 mg ml^−1^ (226 μm), FERM^332^ at 5.71 mg ml^−1^ (157 μm), and FERM^344^ at 9.86 mg ml^−1^ (255 μm). 45 μl of each centrifuged sample were loaded onto a Superdex 200 Increase 3.2/300 column (GE Healthcare), previously equilibrated in 20 mm MES, pH 6.8, 300 mm NaCl, 1 mm TCEP. The scattering profiles from all the proteins were collected in 620 3-s frames.

SAXS data analysis was performed with both PRIMUS (34), from the ATSAS suite (64), and ScÅtter (65). Useful data range for each data set was determined with SHANUM (66). Ambiguity was measured with AMBIMETER (67), and *R_g_* in reciprocal space was calculated with Guinier approximation (68), whereas *R_g_* in real space was derived from pair distribution function *P*(*r*). *P*(*r*) functions and estimation of the *D*_max_ were measured with GNOM (69). CRYSOL (70) was used to calculate, based on FERM^344^ and model-derived FERM^278^, FERM^304^, and FERM^332^ structures, the theoretical scattering profile for each FERM variant and to fit it to the experimental scattering curves. Partitioning of FERM^344^ crystal structure into pseudodomains based on predicted protein dynamics was done with PARCOOR (71), whereas fitting of the partitioned FERM^344^ structures into the scattering data was performed with SREFLEX (71).

## Data availability

Model coordinates and relative structure factors for IDOL FERM^344^ and IDOL F3ab subdomain have been deposited at the Protein Data Bank in Europe (PDBe) database with accession codes 6QLY and 6QLZ, respectively. Other data will be stored in the NKI repository, available upon request from Titia Sixma (t.sixma@nki.nl).

## Supplementary Material

Supporting Information
