# Supplementary material for: Structural analysis of the LDL receptor–interacting FERM domain in the E3 ubiquitin ligase IDOL reveals an obscured substrate-binding site
Source: J Biol Chem. 2020 Jul 29;295(39):13570–83. doi: 10.1074/jbc.RA120.014349 (PMC7521653; doi:10.1074/jbc.RA120.014349)
Supplement: Supporting Information [file supp_295_39_13570__index.html]

Structural analysis of the LDL receptor-interacting FERM domain in the E3 ubiquitin ligase IDOL reveals an obscured substrate binding site — Crystal structures of the IDOL FERM domain — Structural analysis of the LDL receptor–interacting FERM domain in the E3 ubiquitin ligase IDOL reveals an obscured substrate-binding site — Crystal structures of the IDOL FERM domain — Supporting Information 

# Structural analysis of the LDL receptor–interacting FERM domain in the E3 ubiquitin ligase IDOL reveals an obscured substrate-binding site

## Supporting Information

- Supporting Information (to be published online) - supporting figures 1-5
