# Supplementary material for: Structural analysis of the LDL receptor–interacting FERM domain in the E3 ubiquitin ligase IDOL reveals an obscured substrate-binding site
Source: J Biol Chem. 2020 Jul 29;295(39):13570–83. doi: 10.1074/jbc.RA120.014349 (PMC7521653; doi:10.1074/jbc.RA120.014349)
Supplement: Supporting Information [file supp_RA120.014349_160821_2_supp_567043_qdq14c.pdf]

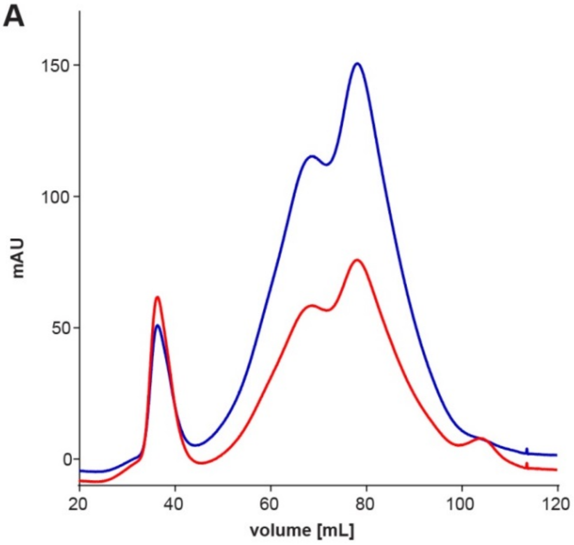

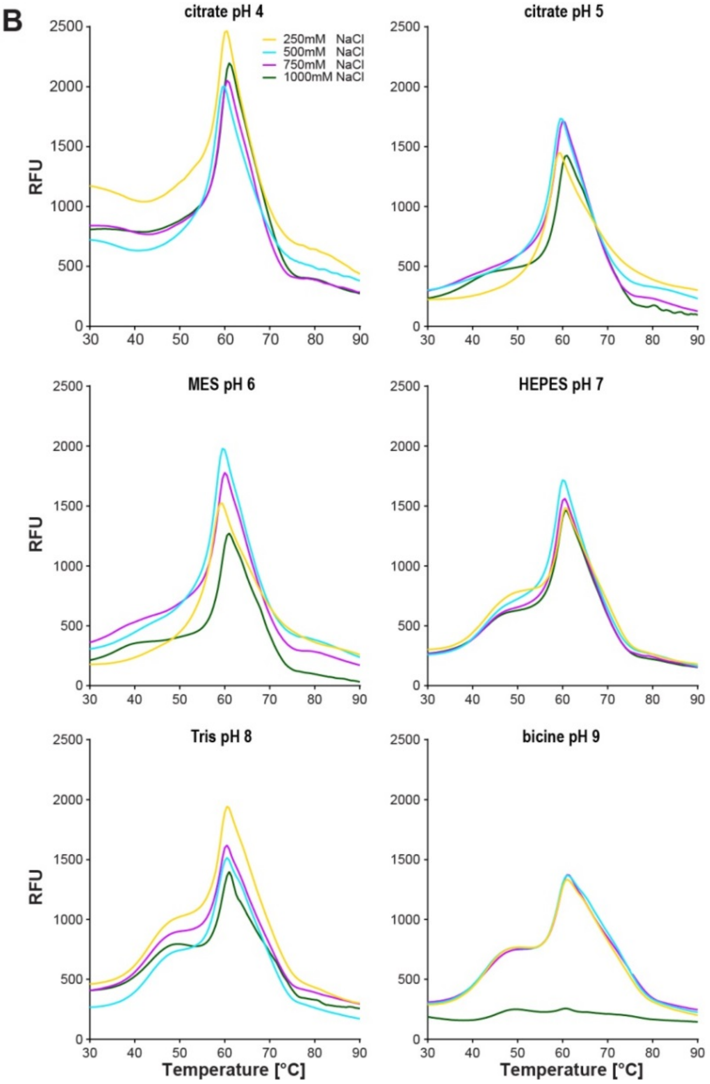

C

	20mM MES pH 6	20mM Tris pH 8
**50mM NaCl**	oligomers	oligomers
**250mM NaCl**	tetramer/dimer	tetramer/dimer
**500mM NaCl**	dimer	trimer/monomer
**1000mM NaCl**	dimer	oligomers/dimer

Supplemental Figure S1. IDOL characterization. (A) Chromatographic profile IDOL. (B) IDOL fluorescence emission spectra under different buffer and salt concentrations. (C) [Table] MALLS MW determination for IDOL under different buffer and salt concentration conditions.

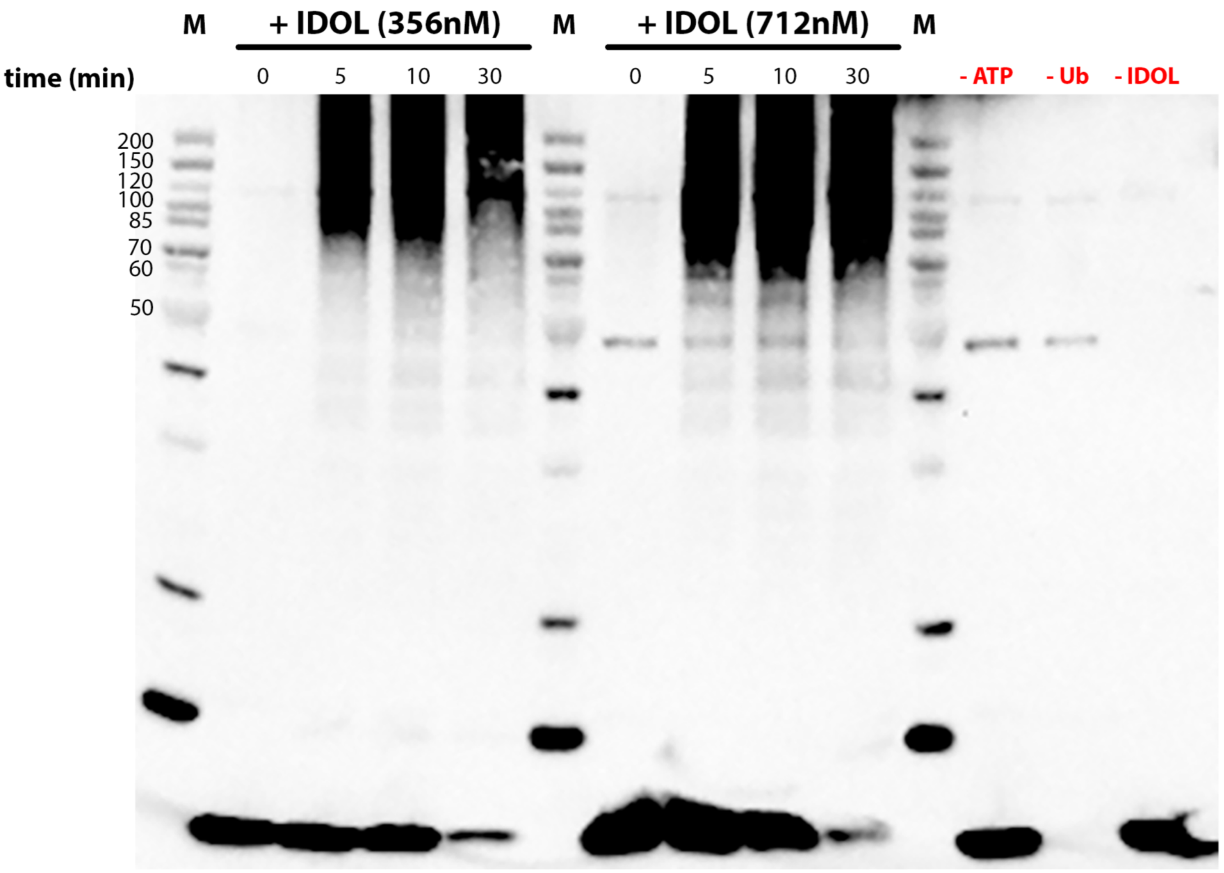

Supplemental Figure S2. *In vitro* IDOL activity assay, showing that ubiquitination activity is dependent on the presence of IDOL and ATP. Detection by immunoblotting for ubiquitin.

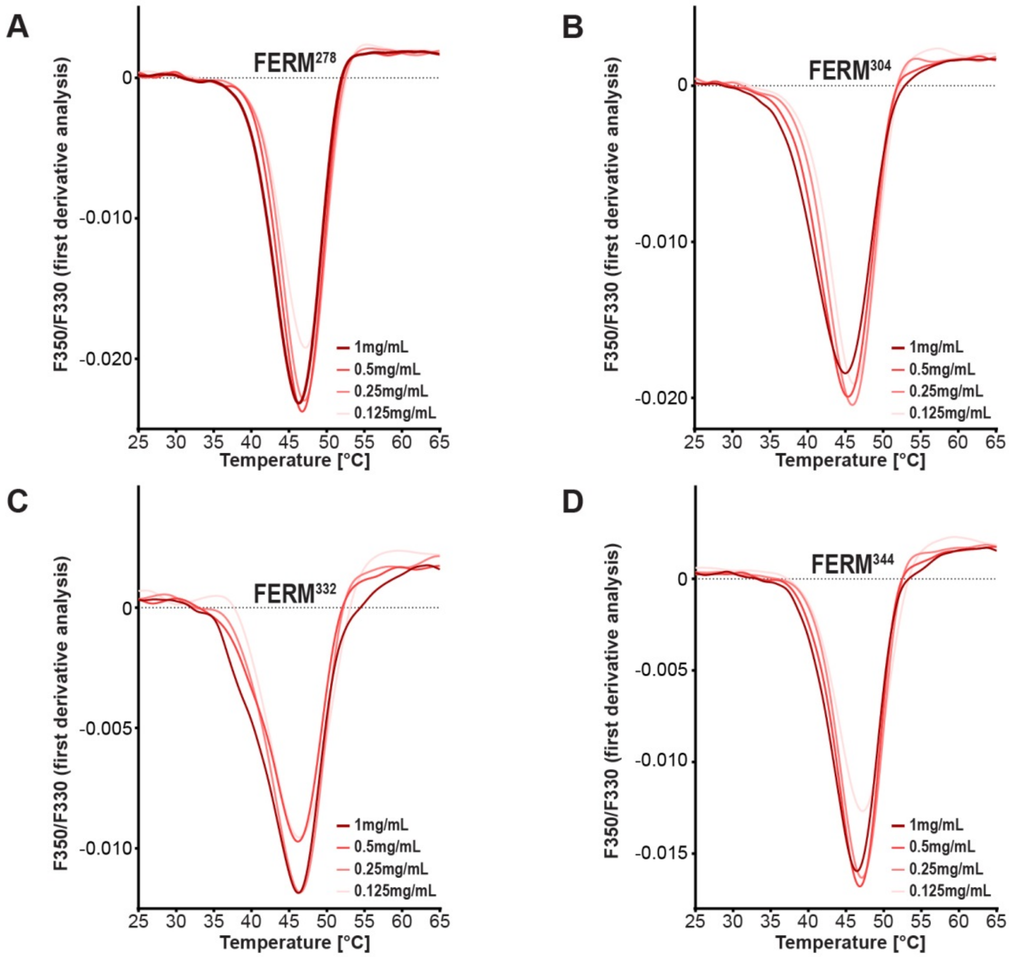

E

	FERM^{278}				FERM^{304}				FERM^{332}				FERM^{344}			
concentration (mg/mL)	1	0.5	0.25	0.125	1	0.5	0.25	0.125	1	0.5	0.25	0.125	1	0.5	0.25	0.125
Tm (°C)	46.1	46.6	46.9	47.0	44.8	45.2	45.7	46.1	45.9	45.9	46.2	46.3	46.3	46.6	46.9	47.1
averaged Tm (°C)	46.7				45.5				46.1				46.7			

Supplemental Figure S3. Thermal stability of the FERM variants. First derivative analysis of F350/F330 fluorescence ratio versus temperature of (A) FERM^{278}, (B) FERM^{304}, (C) FERM^{304} and (D) FERM^{344}. (E) Summary of FERM variants Tm. Determination of Tm for all the FERM variants displays a small standard deviation between experiments done at different concentrations.

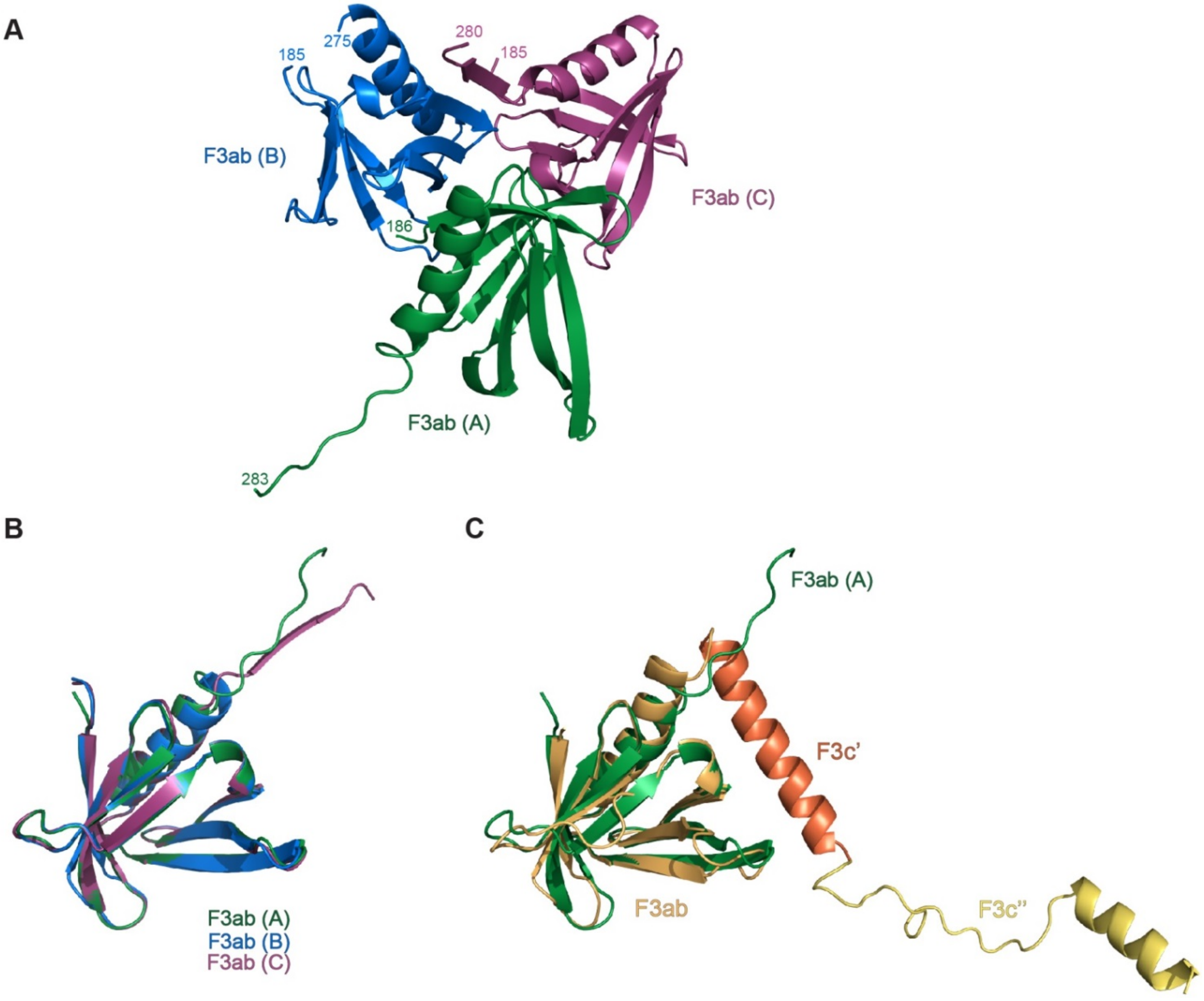

Supplemental Figure S4. Crystal structure of F3ab domain. (A) F3ab asymmetric unit (three copies with three different C-terminal tails). (B) Structural superposition of the three F3ab monomers. (C) Structural comparison between isolated F3ab subdomain (chain A) and FERM^{344} structure.

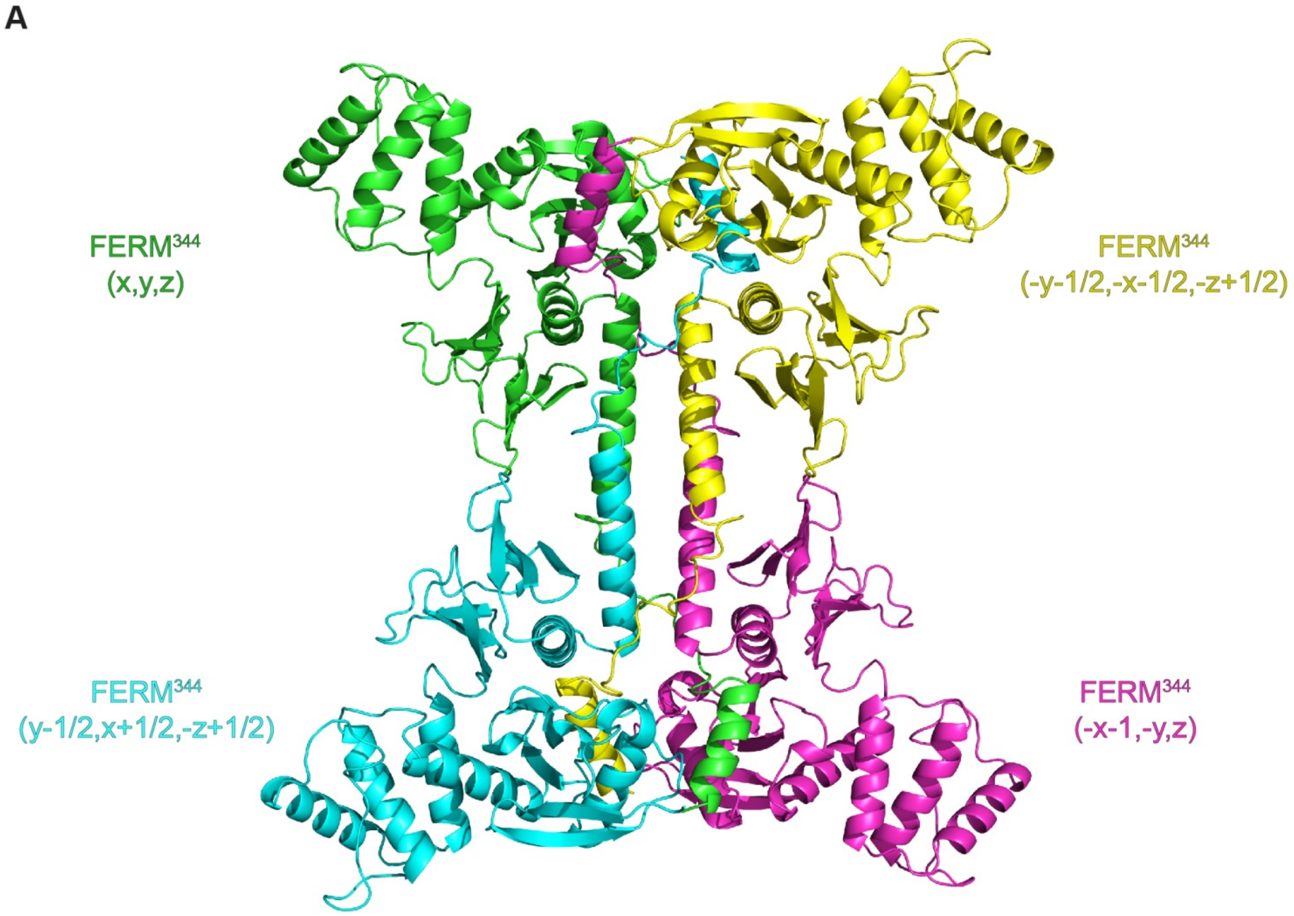

Supplemental Figure S5. FERM[344] crystallographic tetrameric organization. Cartoon representation of FERM[344] monomer (in green) and symmetry related monomer1 (in magenta, generated by symmetry operation -x-1,-y,z), monomer2 (in cyan, generated by symmetry operation y-1/2,x+1/2,-z+1/2) and monomer3 (in yellow, generated by symmetry operation -y-1/2,-x-1/2,-z+1/2).
